# IL‐2 modulates Th2 cell responses to glucocorticosteroid: A cause of persistent type 2 inflammation?

**DOI:** 10.1002/iid3.249

**Published:** 2019-04-17

**Authors:** Tharsan Kanagalingam, Lauren Solomon, Meerah Vijeyakumaran, Nami Shrestha Palikhe, Harissios Vliagoftis, Lisa Cameron

**Affiliations:** ^1^ Department of Pathology and Laboratory Medicine Western University London Ontario Canada; ^2^ Department of Medicine, and Alberta Respiratory Centre University of Alberta Edmonton Alberta Canada

**Keywords:** apoptosis, asthma, IL‐13, IL‐2, steroid, Th2 cells

## Abstract

**Background:**

Glucocorticosteroids (GCs) are the main treatment for asthma as they reduce type 2 cytokine expression and induce apoptosis. Asthma severity is associated with type 2 inflammation, circulating Th2 cells and higher GC requirements.

**Objective:**

The aim of this study was to assess whether ex vivo production of interleukin 2 (IL‐2), a T‐cell survival factor, associated with clinical features of asthma severity, the proportion of blood Th2 cells and Th2 cell responses to GC.

**Methods:**

Peripheral blood from asthma patients (n = 18) was obtained and the proportion of Th2 cells determined by flow cytometry. Peripheral blood cells were activated with mitogen (24 hours) and supernatant levels of IL‐2 and IL‐13 measured by enzyme‐linked immunosorbent assay. In vitro differentiated Th2 cells were treated with dexamethasone (DEX) and IL‐2 and assessed for apoptosis by flow cytometry (annexin V). Level of messenger RNA (mRNA) for antiapoptotic (BCL‐2) and proapoptotic (BIM) genes, IL‐13, GC receptor (GR) and FKBP5 were determined by quantitative real‐time polymerase chain reaction. GR binding was assessed by chromatin immunoprecipitation.

**Results:**

IL‐2 produced by activated peripheral blood cells correlated negatively with lung function and positively with a daily dose of inhaled GC. When patients were stratified based on IL‐2 level, high IL‐2 producers made more IL‐13 and had a higher proportion of circulating Th2 cells. In vitro, increasing the level of IL‐2 in the culture media was associated with resistance to DEX‐induced apoptosis, with more BCL‐2/less BIM mRNA. Th2 cells cultured in high IL‐2 had more IL‐13, less GR mRNA, showed reduced binding of the GR to FKBP5, a known GC‐induced gene, and required higher concentrations of DEX for cytokine suppression.

**Conclusions and Clinical Relevance:**

IL‐2 downregulates Th2 cell responses to GC, supporting both their survival and pro‐inflammatory capacity. These results suggest that a patient's potential to produce IL‐2 may be a determinant in asthma severity.

## 1 INTRODUCTION

Asthma is a syndrome characterized by symptoms of diverse pathogenesis.^1^, ^2^ Type 2 cytokines promote many of the processes responsible for the development of asthma and symptom manifestation. Interleukin 4 (IL‐4) is essential for helper T cell (Th2) cell differentiation and drives B‐cell isotype switching to immunoglobulin E (IgE); IL‐5 is a differentiation factor for eosinophils and mediates their egress from the bone marrow during allergic responses; IL‐13 mediates airway hyperresponsiveness through inflammatory cell infiltration, smooth muscle contraction, and epithelial secretions^3^. These cytokines are produced by both type 2 CD4^+^ Th2 cells^4^, ^5^ and group 2 innate lymphoid cells (ILC2s).^6^ Th2 cells are allergen‐specific memory T cells that circulate between the lymph nodes and periphery and as such can rapidly infiltrate tissues upon allergen exposure.^7^, ^8^, ^9^ While both cell types contribute to overall type 2 cytokine levels, the immunologic memory provided by Th2 cells is considered to mediate the persistent nature of allergic inflammation.^8^, ^10^

Glucocorticoids (GCs) are the main treatment for asthma.^11^ Their efficacy is considered, in large part, to be due to their ability to suppress type 2 cytokines, in vivo,^12^ ex vivo^13^ and in vitro.^14^ In most cases, inhaled GC therapy is sufficient to achieve asthma control, though the amount required varies amongst patients.^15^ In severe asthma, even high doses of inhaled GC fail to control symptoms and adequately improve poor lung function.^15^ Studies show that moderate/severe asthmatics, despite taking high dose inhaled steroid, experienced improved exacerbation rates, lung function, and eosinophilia after using anti‐Th2 cytokine therapies,^16^, ^17^, ^18^, ^19^ revealing that persistent type 2 inflammation was a factor in their disease severity.

Another anti‐inflammatory effect of GC is their ability to induce apoptosis.^20^ This has been well demonstrated for primary human eosinophils,^21^, ^22^ though for T cells the effects vary based on subset examined. For instance, GCs effectively induce apoptosis of thymocytes,^23^ while memory T cells are less sensitive.^24^, ^25^ Recently, we and others reported higher levels of Th2 cells in the blood^26^ and bronchoalveolar lavage (BAL) of severe compared to nonsevere asthmatics.^27^ As such, asthma severity may be related not only to persistent type 2 cytokine expression but also to the inability of GC therapy to eliminate Th2 cells.

IL‐2 is a T cell growth and survival factor that promotes differentiation of the memory T cell phenotype.^28^ In T‐cell lines, IL‐2 was shown to interfere with the GC receptor (GR) nuclear translocation, reducing signaling^29^ and to inhibit GC‐induced apoptosis.^30^ Single nucleotide polymorphisms in IL‐2 and the IL‐2R are associated with asthma severity,^31^ suggesting the strength of the IL‐2 pathway may regulate GC responsiveness. Despite this work, studies linking IL‐2 levels in patients with GC requirements, immune cell profiles and mechanism of action within human Th2 cells have not been examined. Here, we measured IL‐2 production from peripheral blood cells and assessed its relationship with clinical features of asthma, type 2 inflammation and the effect of this cytokine on GC responses of human Th2 cells.

## 2 METHODS

### 2.1 Subjects

This study was approved by the University of Alberta Human Ethics Review Board (approval number PRO1784). All subjects gave informed consent. Subjects were recruited from the tertiary care Asthma Clinic at the University of Alberta during their initial clinic visit. A history of respiratory health was taken and lung function testing performed to assess forced expiratory volume in 1 second (FEV_1_) and forced vital capacity (FVC). Blood was obtained for complete blood count (CBC), IgE measurement, cellular function, and Th2 cell profiling. Severe asthma was defined as patients on high dose inhaled corticosteroids (ICS, ≥1000 µg/day fluticasone equivalent), a second line controller (long‐acting β agonist, leukotriene modifier/or theophylline) and/or oral corticosteroid (OCS) therapy for more than or equal to 50% of the previous year who remain uncontrolled despite this therapy.^15^

### 2.2 Peripheral blood cell activation and IL‐2 measurement

Whole blood (1 mL) from asthmatic patients was obtained, diluted 1:1 with RPMI‐1640 and activated with mitogen (phorbol myristate acetate [PMA], 20 ng/mL; ionomycin, 1 μM) for 24 hours at 37°C, similar to methods developed to study human immune response.^32^, ^33^, ^34^ IL‐2 and IL‐13 levels were measured in the supernatants with IL‐2 (DY202‐05; R&D Systems, Minneapolis, MN) and IL‐13 (851.630.005; Diaclone, Besançon, France) enzyme‐linked immunosorbent assay. All samples were run in duplicate, had low variability between replicates and were all within the standard curve of the assay. The lower limit of detection for IL‐2 was 1.7 pg/mL and IL‐13 was 3.1 pg/mL.

### 2.3 Fluorescence‐activated cell sorting

#### 2.3.1 Profiling of peripheral blood cells

The proportion of helper T cells and Th2 cells in peripheral blood were identified by whole blood staining as in Ref.^26^ In brief, antibodies against CD4 (Clone 1F6; Serotec, Oxford, UK) and CRTh2 (clone BM16; Miltenyi Biotech, San Diego, CA) were used to determine the proportion of cells exhibiting positive staining for these markers, as assessed by flow cytometry. The proportion of helper T cells was identified by low side scatter (SSC^low^) and high CD4^(high)^. Th2 cells were (SSC^low^), CD4^(high)^ and CRTh2 positive and reported as a proportion of total white blood cells (WBC). Flow cytometry data were collected on BD LSR Fortessa using FACSDiva software (Becton, Dickinson and Company, Franklin Lakes, NJ). Gates were set in accordance with the profiles of the isotype control and/or negative control beads.

#### 2.3.2 Apoptosis

Both primary Th2 cells and CCRF‐CEM (ATCC® CCL‐119™, Homo sapiens, T lymphoblast) cells were assessed for apoptosis and cell death using Annexin V and 7‐AAD staining (Biolegend, San Diego, CA). Cells (0.5 × 10^6^) were washed with 1 mL of fluorescence‐activated cell sorting buffer (phosphate‐buffered saline [PBS], 0.5% bovine serum albumin, 0.1% sodium azide, 3% fetal bovine serum [FBS]) then pelleted at 4°C. Cells were then resuspended in 100 μL of annexin V binding buffer (10 mM 4‐(2‐hydroxyethyl)‐1‐piperazineethanesulfonic acid [HEPES] pH 7.4, 140 mM NaCl, 2.5 mM CaCl_2_), and stained with 1 μL of Alexa Fluor 647 Annexin V (640911; BioLegend) and 5 μL of 7‐AAD viability staining solution (BioLegend). Data were acquired using an LSR II (Becton, Dickinson and Company) and analyzed with Flowjo (Version 10; FlowJo, LLC, Ashland, OR). Data are reported as the proportion of the total cell population.

### 2.4 Cell culture

#### 2.4.1 Primary human Th2 cells

Peripheral blood mononuclear cells from healthy donors were obtained by density centrifugation using Ficoll Histopaque Plus (GE Healthcare, Chicago, IL). As previously described,^35^, ^36^ helper T cells were isolated by negative selection with a CD4^+^ T cell Isolation Kit II (Miltenyi Biotech, San Diego, CA) and cultured in X‐Vivo 15 media (Lonza, Walkersville, MD) supplemented with 10% FBS (Wisent, Saint‐Jean‐Baptiste, QC, Canada), 1× penicillin‐streptomycin‐glutamine (Gibco, Invitrogen, Thermo Fisher Scientific, Waltham, MA). For Th2 differentiation, CD4^+^ T cells were activated for 3 days on plate‐bound antibody (α) against CD3 (clone UCHT1, 1 μg/mL) and αCD28 (clone 37407, 1 μg/mL) in the presence of recombinant human rhIL‐2 (5 ng/mL; R&D Systems Inc), rhIL‐4 (10–20 ng/mL; R&D Systems Inc), blocking antibody for interferonγ (IFNγ) (AF‐285‐NA, 1 µg/mL, R&D Systems Inc) and IL‐12 (Clone C8.6, 1 µg/mL, R&D Systems Inc). After activation, cells were proliferated with the same concentration of cytokines (IL‐2 and IL‐4) and blocking antibodies (IFNγ and IL‐12) for 4 days. Following two rounds of this differentiation protocol, Th2 cells (CRTh2^+^CD4^+^, purity of ~98%) were obtained using a CRTh2 isolation kit (Miltenyi Biotech, San Diego, CA). Th2 cells were maintained on cycles of IL‐2 and plate‐bound αCD3/αCD28 (3 days) or just IL‐2 (4 days) at 2 × 10^6^ cells/mL. For experiments, Th2 cells (1.3 × 10^6^ cells/mL) were examined following exposure to various concentrations of dexamethasone (DEX; Sigma‐Aldrich) and IL‐2.^13^, ^37^

#### 2.4.2 Immortalized T cells

CCRF‐CEM cells (clone CRM‐CCL‐119) are a CD4^+^ T cell line derived from lymphocytic leukemia and were purchased from American Type Culture Collection (VA). Cells were grown in RPMI‐1640 media (Sigma‐Aldrich) supplemented with 10% FBS (Hyclone Scientific, Fisher Scientific, ON, Canada) and 1× penicillin‐streptomycin‐glutamine (Gibco, Invitrogen, Thermo Fisher Scientific). Cells were incubated at 37°C, in 85% humidity and 5% CO_2_. Cells were maintained at 0.2 × 10^6^cells/mL and were re‐seeded every 2 days.

### 2.5 Quantitative real‐time polymerase chain reaction

Total RNA was isolated using the RNeasy mini plus extraction kit (Qiagen, ON, Canada) and complementary DNA was synthesized from 400 ng RNA using iScript reverse transcriptase (Bio‐Rad, Hercules, CA). TaqMan gene expression assays (Life Technologies, Carlsbad, CA) for IL‐13 (Hs00174379_m1), B‐cell lymphoma 2 (BCL‐2; Hs00608023_m1), Bcl‐2 interacting mediator (BIM; Hs00708019_s1), total GC receptor (GR; Hs00353740_m1), GRβ (GRβ; (Hs00354508_m1), FK506 binding protein 51 (FKBP5; Hs01561006_m), and glyceraldehyde 3‐phosphate dehydrogenase (GAPDH; Hs02786624_g1) were used. Data were analyzed using cycle threshold (*C*_t_) relative to housekeeping gene GAPDH. Fold increase relative to the control condition was assessed for experimental treatments using $2^{-\Delta \Delta C_{t}}$.

### 2.6 Chromatin immunoprecipitation

Primary Th2 cells were fixed (1% formaldehyde, 10 minutes) followed by the addition of glycine (0.125M final concentration, 5 minutes). Cells were washed in ice‐cold PBS (3×) and snap‐frozen in pellets (20 × 10^6^ cells). Pellets were lysed in chromatin immunoprecipitation (ChIP) lysis buffer (10 mM piperazine‐N,N′‐bis(2‐ethanesulfonic acid) [PIPES], 10 mM KCl, 0.5% NP40, 0.1 mM ethylenediaminetetraacetic acid [EDTA] and EGTA [ethylene glycol‐bis(β‐aminoethyl ether)‐*N*,*N*,*N*′,*N*′‐tetraacetic acid]) with 1 mM dithiothreitol and 0.5 mM phenylmethylsulfonyl fluoride (PMSF) on ice (15 minutes). Nuclei were pelleted and lysed in ChIP nuclear lysis buffer (50 mM Tris‐Cl, 10 mM EDTA, 1% sodium dodecyl sulfate [SDS]) with leupeptin and aprotinin (0.02 μg/mL each) and PMSF (0.5 mM, on ice 10 minutes). The lysate (0.5 mL) was sonicated on BioRuptor (35 cycles, 30 seconds per pulse, 30 seconds cooling between pulses; Diagenode) and input sample collected. Protein G Dynabeads (Invitrogen, CA) were washed in PBS‐T and preincubated with 10 μg target antibody (20 minutes, room temperature with rotation). Antibodies were: mouse anti‐human GR (G‐5) (sc‐393232; Santa Cruz Biotechnology, Dallas, TX) and mouse immunoglobulin G (IgG) (M5284; Millipore, Burlington, MA). Beads were washed in PBS‐T (200 μL) and incubated overnight with 100 μL sonicated chromatin in 900 μL immunoprecipitation buffer (16.7 mM Tris‐HCL, 167 mM NaCl, 1.2 mM EDTA, 1% Triton‐X100, 0.01% SDS) at 4˚C with rotation. Beads were washed with 1 mL each: low salt wash buffer (2 mM Tris pH8, 1% Triton‐X, 1% SDS, 167 mM NaCl) (1×), High Salt Wash Buffer Buffer (2 mM Tris pH8, 1% Triton‐X, 0.1% SDS, 2 mM EDTA, 0.5 M NaCl) (1X), LiCl wash buffer (10 mM Tris pH 8, 0.25M LiCl, 1% NP‐40, 1% sodium deoxycholate, 1 mM EDTA) (1×) and TE buffer (10 mM Tris, pH 8, 1 mM EDTA) (1×). Chromatin was eluted with 150 μL 1% SDS and 0.1M NaHCO3 (3×; 15 minutes) and crosslinks were reversed with 0.2 M NaCl (overnight, 65˚C). DNA was purified using a QIAquick PCR Purification Kit (Qiagen, Hilden, Germany). Quantitative polymerase chain reaction was performed using SsoFast EvaGreen Supermix (Bio‐Rad). Primers used were for FKBP5 (F‐TAACCACATCAAGCGAGCTG; R‐GCATGGTTTAGGGGTTCTTG). Negative control for ChIP was IP using mouse IgG. Data were analyzed using ΔΔ*C*_t_ method. Δ*C*_t_ for each sample was calculated as *C*_t__input_−*C*_t__pulldown_ and the $2^{-\Delta \Delta C_{t}}$was the Δ*C*_t_ of IgG subtracted from Δ*C*_t_ of target antibody. Data are presented relative to vehicle control.

The GEO SuperSeries accession number GSE36890^38^ was retrieved for evaluation of Signal transducer and activator of transcription 5 (STAT5) binding to BCL‐2 and IL‐13 loci. STAT5A binding in wild‐type control and IL‐2 treated splenocytes was retrieved as.bed.gz file and converted to BigWig for viewing in the UCSC Genome Browser.

### 2.7 RNA sequencing

Total RNA was isolated from using a Qiagen RNeasy Plus Kit, eluted in 30 μL RNAse‐free water and concentration determined (Nanodrop Quantification). Bioanalysis of RNA was conducted using an Agilent Bioanalyzer 2100 (Agilent, Santa Clara, CA). rRNA‐depleted (HMR) stranded libraries were produced using the services of Genome Quebec. Libraries were multiplexed and sequenced on an Illumina HiSeq 4000 (Illumina, San Diego, CA) in PE100 mode. Paired‐end FASTQ files were evaluated for overall quality using FastQC Read Quality reports (Galaxy Version 0.72 www.usegalaxy.org). Sequencing adapters were removed from paired‐end files using IlluminaClip in trimmomatic.^39^ Maximum mismatch count = 2 and sliding window trimming Nbases = 4, AVGQual = 20. Trimmed reads were aligned to Hg38 using HiSat2 (Version 2.1.0) in fr mode. No‐mixed/no‐discordant behavior disabled. Gene expression in RNA‐Seq experiments was determined using featureCounts^40^ (Version 1.6.2) in stranded (forward) mode, using Gene‐ID features from Gencode.v29.annotation GFF feature type filter—exon, disabled multimapping, minimum mapping quality‐10. Gene expression in RNA‐Seq experiments was determined using featureCounts^40^ (Version 1.6.2) in stranded (forward) mode, using Gene‐ID features from Gencode.v29.annotation GFF feature type filter—exon, disabled multimapping, minimum mapping quality‐10. RNAseq data are available upon request.

### 2.8 Statistical analysis

Patients were stratified by median IL‐2 (42 600 pg/mL) as in Refs.^34^, ^41^ Receiver operator curve (ROC) analyses were also used as in Ref.^42^ using FEV_1_ less than 80% predicted or taking more than or equal to 1000 μg/day inhaled steroid and gave a similar cut‐point (42 993 pg/mL; FEV_1_: Sen = 0.77, Spec = 0.77; Inhaled Steroid: Sen = 0.8, Spec = 0.62). Stratified data were analyzed for statistical significance using Pearson's the χ^2^ test for categorical data and independent *t* test for continuous variables. Correlations were determined using Pearson's or Spearman's correlation, depending on the normality of the data. For cell culture experiments, statistical significance for apoptosis and gene expression were determined by analysis of variance with post hoc analysis (Student‐Newman‐Keuls method) or Student *t* test. Data were analyzed using SigmaPlot Version 12.5 and considered significant with *P* < 0.05.

### 2.9 Ethical approval

This study was approved by the University of Alberta Human Ethics Review Board (approval number PRO1784) and the Western University Health Science Research Ethics Board (approval number 106770). All subjects gave informed consent.

## 3 RESULTS

### 3.1 Peripheral blood cell production of IL‐2 associates with asthma severity

**Figure 1:**
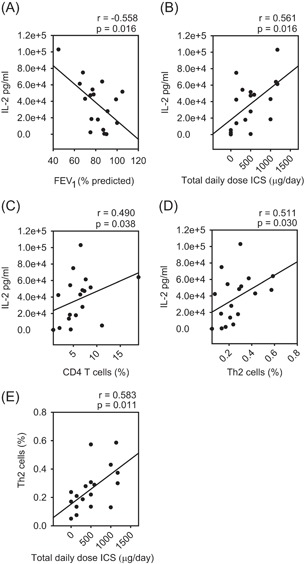
IL‐2 production associates with asthma severity. A, IL‐2 production was inversely correlated with FEV_1_ (% predicted) and (B) positively correlated with total daily dose of inhaled corticosteroid (fluticasone equivalent μg/day). IL‐2 production was positively correlated with the proportion of circulating (C) CD4^+^ T cells and (D) Th2 cells. E, Proportion of circulating Th2 cells were positively correlated with total daily dose of inhaled corticosteroid. FEV1, forced expiratory volume in 1 second; ICS, inhaled corticosteroid; IL‐2, interleukin 2; Th2, T helper cell

Patients were recruited (n = 18) from new referrals to our Asthma Center and clinically characterized to assess asthma severity. The amount of IL‐2 produced by activated peripheral blood cells was variable, ranging from 14 to 102 933 pg/mL. Patients stratified based on median IL‐2 production (42 600 pg/mL) showed no difference in age, body mass index, IgE levels or smoking status. However, those with high IL‐2 (>median) did have lower FEV_1_ and were taking a higher daily dose of inhaled corticosteroid (Table 1). Analyzing the whole population together, IL‐2 production was inversely correlated with FEV_1_ (*r* = −0.558, *P* = 0.0162; Figure 1A) and positively correlated with total daily dose of inhaled corticosteroid (*r* = 0.561, *P* = 0.0155; Figure 1B), suggesting its association with asthma severity. While this study population consisted of only four severe asthmatics, our analysis does suggest this group is characterized by higher IL‐2 production (56 380 pg/mL) compared to nonsevere asthmatics (32 133 pg/mL, *P* = 0.07). Further, when patients were stratified based on the daily dose of inhaled steroid, those taking more than or equal to 1000 μg/day had higher IL‐2 production (Table 2).

**Table 1:** Clinical characteristics of asthmatics stratified by IL‐2

IL‐2, pg/mL	<42 600^a^		*P* value
Number	9	9	
IL‐2 (mean, pg/mL)	14 221	60 822	
Sex (% F)	67	44	NS
Age	42.1	38.9	NS
Body mass index (BMI)	31.97	37.76	NS
IgE (kU/L)	1.86	0.16	NS
History of smoking (%)	44.4 (4/9)	44.4 (4/9)	NS
Pack year	2.94	7.88	NS
Currently smoking (%)	0	0	NS
FEV_1_ ^b^(% Pred)	87.6	74.3	**0.047**
FEV_1_/FVC^c^	71.22	64.77	NS
Total daily dose ICS^d^ (μg/day)	293.8	722.3	**0.026**
Oral corticosteroid (%)	11	11	NS
Short‐acting β agonist (%)	44	22	NS
Long‐acting β agonist (%)	78	56	NS
Montelukast (%)	22	0	NS
Severe asthma (%)^e^	11	22	NS
Eosinophils (%)^g^	4.08	3.17	0.705
Neutrophils (%)^g^	60.8	58.28	0.575
% CD4^+^ T cells	4.54	7.98	0.070
% CD4^+^ CRTh2^+^	4.687	4.652	NS
% CD4^+^ CRTh2^+^/WBC^f^	0.167	0.353	**0.005**
IL‐13 (pg/mL)	306.8	584	**0.042**

Abbreviations: ICS, inhaled corticosteroids; IgE, immunoglobulin E. Bold text denotes *p* value <0.05.

^a^ Data stratified by median IL‐2 (42 600 pg/mL).

^b^ Forced expiratory volume in 1 second.

^c^ Forced vital capacity.

^d^ ICS, inhaled corticosteroid, fluticasone equivalent.

^e^ ATS/ERS Guidelines.^15^

^f^ WBC, peripheral white blood cells.

^g^ Percent of complete blood count.

**Table 2:** Clinical characteristics of asthmatics stratified by ICS^a^

	ICS^a^ (total μg/day)*		
ICS^a^ (total μg/day)	<1000	>1000	
Number	13	5	***P*** **value**
Total daily dose ICS^a^ (μg/day)	282	1097	**7.1E−07**
Sex (% F)	62 (8/13)	40 (2/5)	0.440
Age	41.5	38.0	NS
Body mass index (BMI)	39.58	39.17	NS
IgE (kU/L)	114.6	521.8	0.076
History of smoking (%)	38	60	NS
Pack year	2.8	12.2	**0.031**
Currently smoking (%)	0	0	NS
FEV_1_ ^c^(% Pred)	85.8	68.4	**0.016**
FEV_1_/FVC^b^	70.8	60.6	0.093
Oral corticosteroid (%)	8.0	20	NS
Short‐acting β agonist (%)	20	20	NS
Long‐acting β agonist (%)	54	100	NS
Montelukast (%)	8	20	NS
Severe asthma (%)^d^	0	80	**0.001**
Eosinophils (%)^f^	4.04	2.56	NS
Neutrophils (%)^f^	61.85	53.73	NS
% CD4^+^ T cells	5.31	8.77	**0.053**
% CD4^+^ CRTh2^+^	4.74	4.49	NS
% CD4^+^ CRTh2^+^/WBC^e^	0.22	0.36	**0.036**
IL‐13 (pg/mL)	408.74	540.97	0.418
IL‐2 (pg/mL)	29, 687.74	57 890.33	**0.030**

Abbreviations: ICS, inhaled corticosteroids; IgE,immunoglobulin E; WBC, white blood cells. Bold text denotes *p* value <0.05.

* Data stratified by 1000 μg/day.

^a^ ICS, inhaled corticodsteroid, fluticasone equivalent.

^b^ FVC, forced vital capacity.

^c^ FEV_1_, forced expiratory volume in 1 second.

^d^ ATS/ERS Guidelines.^15^

^e^ WBC, peripheral white blood cells.

^f^ Percent of complete blood count.

### 3.2 Peripheral blood cell production of IL‐2 associates with type 2 inflammation

The propensity for high IL‐2 production was also related to the degree of type 2 inflammation. Supernatants from patients with high IL‐2 following activation of their peripheral blood cells contained 1.9‐fold more IL‐13 (584.1 vs 306.8; Table 1) and flow cytometry staining of whole blood showed these patients had higher proportions of CD4^+^ T cells (7.99 vs 4.55) and Th2 cells (0.35 vs 0.17; CD4^+^CRTh2^+^ T cells as a proportion of total white blood cells; Table 1). IL‐2 production correlated with the proportion of both CD4^+^ T cells (Figure 1C, *r*_s_ = 0.490, *P* = 0.038) and Th2 cells (Figure 1D, *r*_s_ = 0.511, *P* = 0.030). Th2 cells also correlated with total daily dose of inhaled corticosteroid (Figure 1E, *r* = 0.583, *P* = 0.011).

### 3.3 The IL‐2‐GC axis regulates survival of Th2 cells

**Figure 2:**
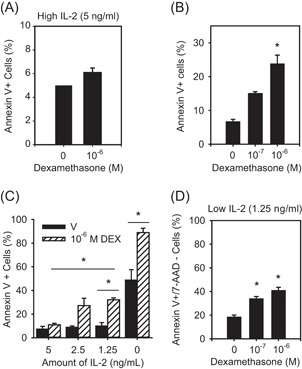
IL‐2 influences Th2 cell sensitivity to GC‐induced apoptosis. A, Th2 cells cultured in 5 ng/mL of IL‐2 did not exhibit an increase in annexin V^+^ cells in response to DEX (10^−6^M; n = 4). B, CCRF‐CEM cells, an immortalized T‐cell line, showed an increase in annexin V^+^ cells in response to DEX (10^−6^M; n = 4). C, Titration of IL‐2 revealed that the proportion of Th2 cells staining positive for annexin V increased significantly with lower concentrations of IL‐2 (n = 3). D, Th2 cells cultured in low IL‐2 (1.25 ng/mL) exhibited a significant increase in apoptosis annexin V+/7AAD− cells and responded to 10‐fold less DEX (n = 3). Data are presented as mean ± standard error.*P < 0.05 vs vehicle (ie, no DEX). DEX; dexamethasone; GC, glucocorticosteroid; IL‐2, interleukin 2; Th2, T‐helper cell; V, vehicle

GCs induce apoptosis and cell death of various T cell subsets, though to our knowledge no studies have investigated their effect on human Th2 cells. Therefore, we assessed in vitro differentiated primary human Th2 cell response to DEX (10^−6^M, 48 hours). Interestingly, we failed to see any increase in annexin V^+^ cells (Figure 2A), indicating there was no effect on apoptosis and/or cell death. This was surprising since this level of DEX was able to induce apoptosis in an immortalized T cell line (CCRF CEM, Figure 2B). A major difference between these two experiments was that the primary Th2 cells were cultured in IL‐2 (5 ng/mL). Considering that patients with high IL‐2 production had more Th2 cells (Table 1), we next assessed whether titrating the level of this growth factor would alter Th2 cell sensitivity to GC‐induced apoptosis. When Th2 cells were cultured with DEX and lower concentrations of IL‐2 a significant increase in annexin V^+^ cells was observed (Figure 2C). Indeed, Th2 cells showed sensitivity to 10‐fold less DEX (10^−7^M) when cultured with low IL‐2 (1.25 ng/mL; Figure 2D) compared to high IL‐2 (5 ng/mL; Figure 2C).

**Figure 3:**
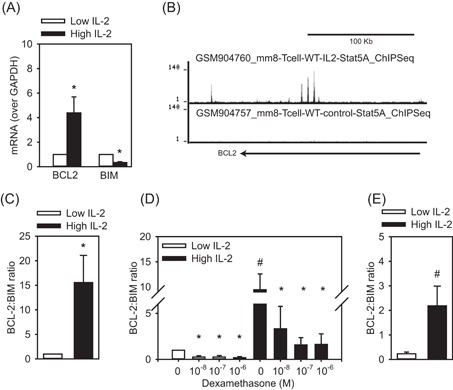
IL‐2 influences the BCL‐2 Rheostat. A, mRNA for BCL‐2 was elevated and BIM was reduced when Th2 cells were cultured in high IL‐2 (5 ng/mL) compared to low IL‐2 (1.25 ng/mL). B, Genome browser tracks for STAT5 ChIP under control and IL‐2 replete conditions within the BCL‐2 gene body. C, High IL‐2 increases the BCL‐2:BIM ratio (n = 7). D, The BCL‐2:BIM ratio remains strong (above 1) even after DEX treatment (24 hours, n = 3). E, Th2 cells cultured in high IL‐2 and DEX had greater BCL‐2:BIM ratios than low IL‐2 and DEX (10^−8^–10^−6^M combined, n = 9). Data are presented as mean ± standard error. *P < 0.05 vs no DEX (for each IL‐2 concentration); ^#^ *P* < 0.05, high vs low IL‐2. ChIP, chromatin immunoprecipitation; DEX, dexamethasone; IL‐2, interleukin 2; mRNA, messenger RNA; STAT5, signal transducer and activator of transcription 5; Th2, T‐helper cell

Apoptosis is regulated by the expression of proapoptotic and antiapoptotic genes of the BCL‐2 family, with the balance between these referred to as the BCL‐2 rheostat.^43^, ^44^ Since IL‐2 induces the antiapoptotic gene BCL‐2^45^ and GC induces the proapoptotic gene BIM,^46^ we assessed the expression of these factors. Th2 cells cultured in high IL‐2 had more BCL‐2 and less BIM mRNA compared to Th2 cells cultured in low IL‐2 (Figure 3A). This is likely a transcriptional effect since we found (using GSE36890^38^) that IL‐2 treatment of murine splenocytes mediates STAT5 binding the BCL‐2 locus (Figure 3B). The ratio of BCL‐2 to BIM (BCL‐2:BIM) was significantly higher in Th2 cells cultured in high vs low IL‐2 (Figure 3C) and remained higher following DEX treatment (Figure 3D). Indeed, when the data for all three DEX concentrations were combined (10^−8^–10^−6^M), the BCL‐2:BIM ratio of Th2 cells cultured with high vs low IL‐2 was significantly higher (Figure 3E), suggesting that it regulates Th2 cell susceptibility to GC‐induced apoptosis.

### 3.4 The IL‐2‐GC axis regulates the suppression of type 2 cytokines

**Figure 4:**
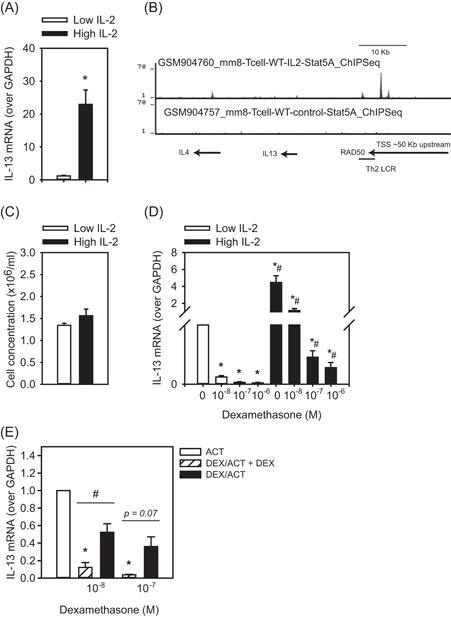
IL‐2 dampens GC‐mediated suppression of IL‐13. A, Th2 cells cultured with high IL‐2 (5 ng/mL) had significantly more IL‐13 mRNA compared to those cultured in low IL‐2 (1.25 ng/mL; n = 6). B, Genome browser tracks for STAT5 ChIP under control and IL‐2 replete conditions within the Th2 locus control region, an enhancer of IL‐13. C, Th2 cells cultured in high or low IL‐2 showed no difference in cell number (n = 6). D, The ability of DEX treatment to suppress IL‐13 mRNA was significantly less when Th2 cells were cultured in high IL‐2 vs low IL‐2 (n = 6). E, Continual exposure to DEX was required for suppression of IL‐13, since Th2 cells pretreated with DEX (24 hours) and then activated (ACT) with PMA/ionomycin (24 hours) in the absence of DEX (DEX/ACT) had significantly more IL‐13 mRNA than Th2 cells receiving DEX both as a pretreatment and during activation (DEX/ACT + DEX; n = 3). Data are presented as mean ± standard error. *P < 0.05 comparison of DEX vs vehicle for each IL‐2 concentration; ^#^ *P* < 0.05, comparison of each DEX concentration high vs low IL‐2. ChIP, chromatin immunoprecipitation; DEX, dexamethasone; IL‐2, interleukin 2; mRNA, messenger RNA; STAT5, signal transducer and activator of transcription 5; Th2, T‐helper cell

Since patients exhibiting high IL‐2 production from peripheral blood cells also produced more IL‐13 (Table 1), we assessed whether IL‐2 directly regulates IL‐13 expression. We found that Th2 cells cultured with high IL‐2 (24 hours) expressed significantly more IL‐13 mRNA compared to those cultured with low IL‐2 (Figure 4A). ChIP‐sequencing analysis showed (GSE36890^38^) that IL‐2 treatment of murine splenocytes mediated STAT5 binding to the Th2 locus control region (Figure 4B), a known enhancer of IL‐13 expression.^47^ Since it did not influence Th2 cell numbers (Figure 4C), this would suggest the IL‐2 effect was due to heightened activation of IL‐13 transcription rather than Th2 cell proliferation.

To assess the effect of IL‐2 on the ability of GC to suppress type 2 cytokines, we cultured Th2 cells with varying concentrations of IL‐2 and DEX. When Th2 cells were cultured in high IL‐2 the ability of DEX to suppress IL‐13 mRNA was significantly less than when cells were cultured with low IL‐2 (Figure 4D). This did not appear to be due to IL‐2 influencing a shift toward Th17 or Treg phenotypes, as mRNA levels for IL‐17 family members and IL‐10 were low (Table 3). To assess the durability of GC suppression following activation, Th2 cells were pretreated with DEX (24 hours), washed and activated with mitogen in the presence or absence of DEX (24 hours) and high IL‐2. Th2 cells receiving DEX as both a pretreatment and during activation exhibited almost complete suppression of IL‐13 mRNA (88% and 96%, respectively), while cells receiving DEX only as a pretreatment had substantially more IL‐13 mRNA following activation (Figure 4E).

**Table 3:** RNA‐sequencing of Th2 cells

	Vehicle	0.1 µM Dex	*P*
IL2	0.96 ± 0.01	0.72 ± 0.72	NS
IL4	**162.56** ± **31.75**	**57.97** ± **12.53**	**0.014**
IL5	**6105.18** ± **1745.48**	**385.67** ± **72.35**	**0.003**
IL13	**21310.53** ± **7745.81**	**718.97** ± **201.87**	**0.006**
IL10	**17.33** ± **3.60**	**5.37** ± **0.25**	**0.050**
IL17A	ND	ND	
IL17B	1.58 ± 0.61	1.01 ± 0.04	NS
IL17C	ND	ND	
IL17D	29.65 ± 2.56	42.30 ± 8.22	NS

Abbreviations: Dex, dexamethasone; IL, interleukin; ND, not detected; NS, not significant. Bold text denotes *p* value <0.05.

### 3.5 IL‐2 inhibits GR expression and signaling

**Figure 5:**
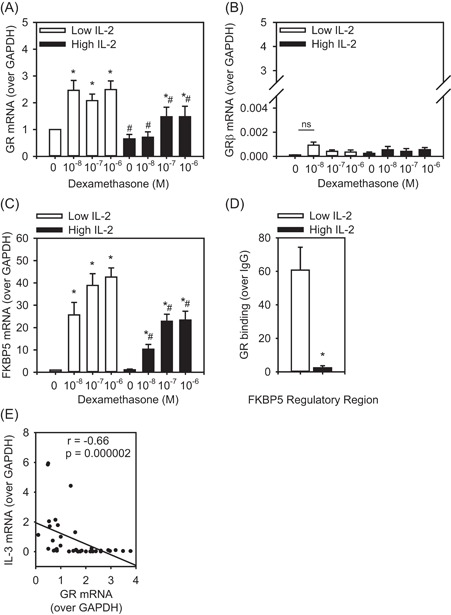
GR abundance and function are decreased by IL‐2. A, Th2 cells cultured with high IL‐2 (5 ng/mL) had significantly less GR mRNA compared to those cultured in low IL‐2 (1.25 ng/mL; n = 6). B, GRβ mRNA was low in Th2 cells and was unaffected by IL‐2 levels. C, FKBP5, a known GC inducible gene, had significantly reduced expression under high IL‐2 high. D, Chromatin immunoprecipitation (ChIP) for binding of GR to a regulatory element within the FKBP5 locus. GR binding was significantly less in Th2 cells cultured in high IL‐2 (5 ng/mL) compared to low IL‐2 (1.25 ng/mL). E, Expression of GR and IL‐13 mRNA were inversely correlated. Data are presented as mean ± standard error. *P < 0.05 comparison of DEX vs vehicle for each IL‐2 concentration; ^#^ *P* < 0.05, comparison of each DEX concentration high vs low IL‐2. DEX, dexamethasone; GR, glucocorticoid receptor; IL‐2, interleukin 2; mRNA, messenger RNA; Th2, T‐helper cell

To examine the mechanism underlying our observations that IL‐2 dampens the ability of GC to induce apoptosis and suppress IL‐13, we assessed expression of the GR, total levels as well as GR beta (β), a dominant negative isoform associated with reduced GC sensitivity.^48^ The data are presented relative to control GR level (vehicle, low IL‐2) and show that total GR mRNA was fairly abundant (~*C*_t_ 27; Figure 5A), while there were extremely low‐to‐no levels of the GRβ isoform (*C*_t_ >37 cycles to not detected; Figure 5B). This result indicates that induction of the dominant negative GRβ isoform is likely not the mechanism underlying our finding of IL‐2 dampening the effects of GC. However, Th2 cells cultured in high IL‐2 had lower levels of total GR mRNA than those cultured in low IL‐2 (Figure 5A). As such, we next assessed if this reduction in GR mRNA was sufficient to influence GR signaling. To do this, we measured FKBP5 expression, a gene known to be induced by GC.^49^ We found that Th2 cells cultured in high IL‐2 had significantly less FKBP5 mRNA (in response to all DEX concentrations) compared to those cultured in low IL‐2 (Figure 5C). We developed a ChIP assay for GR, which demonstrated that Th2 cells cultured in high IL‐2 exhibited significantly less GR binding to a regulatory element within the FKBP5 locus than those cultured in low IL‐2 (Figure 5D). Furthermore, the level of GR mRNA was inversely correlated with IL‐13 mRNA (*r*_s_ = −0.66, *P* = 0.000002; Figure 5E). Collectively, these data suggest that IL‐2 dampening GR signaling resulted in the higher levels of IL‐13 mRNA (Figure 4D).

## 4 DISCUSSION

IL‐2 is elevated in the airways of symptomatic vs asymptomatic asthmatics^50^, ^51^ and in steroid‐resistant vs sensitive asthmatics.^52^ Our study extends these earlier findings by assessing the relationship between peripheral IL‐2, clinical indices of asthma severity and immune cell profile. We found that peripheral blood cell production of IL‐2 correlates with poor lung function, increased GC requirements and heightened type 2 inflammation. In vitro human Th2 cells maintained in high IL‐2 exhibited lower expression of the GR and were less responsive to GC‐induced apoptosis and cytokine suppression. Though our study was not designed to examine steroid‐resistant asthma per se, which involves assessment presteroid/poststeroid treatment as in Ref.^52^ we did observe that patients with higher inhaled steroid requirements produced more IL‐2. Collectively, our results suggest that an environment of high IL‐2 may mediate asthma severity by dampening GC responsiveness, resulting in persistence of Th2 cells and production of type 2 cytokines.

Since IL‐2 is a growth factor known to mediate proliferation of CD4^+^ T cells,^53^ the higher proportion of Th2 cells in those exhibiting high IL‐2 production could be due to increasing CD4^+^ T cell proliferation and/or Th2 cell development.^54^ However, our in vitro data showed that Th2 cells cultured in high IL‐2 exhibited a BCL‐2:BIM ratio above 1 (ie, more BCL‐2 than BIM). Though IL‐2 induction of BCL‐2 is well documented,^45^, ^55^ to our knowledge this is the first report of IL‐2 suppressing BIM expression. Further, our integrated analysis of the BCL‐2:BIM ratio suggests IL‐2 modulates both sides of this rheostat within human Th2 cells, tipping them toward an antiapoptotic phenotype. The level of IL‐2 in the culture media also influenced IL‐13 expression, similar to a previous report with antigen‐specific Th clones.^56^ Both mechanisms may be transcriptional, as we found that IL‐2 induces STAT5 binding to BCL‐2 and IL‐13 loci.

IL‐2 dampened the capacity of GC to induce apoptosis. This may be due to its reducing GR expression and signaling, further shifting the BCL‐2:BIM ratio, as GR binding to the BIM locus, has been shown.^57^ In the case of IL‐13, we found an inverse correlation between GR and IL‐13 mRNA levels, suggesting that the IL‐2 effect on GC‐induced cytokine suppression was also due to reduced GR signaling. Indeed, GR has been shown to interfere with many factors that mediate IL‐13 transcription such as STAT5,^29^, ^54^ proteins within the AP‐1 proteins complex^58^ and nuclear factor‐κB.^59^, ^60^, ^61^ While our results do not rule out other mechanisms influencing GC sensitivity in vivo, such as level of GRβ^48^ and posttranslational modifications that reduce GR nuclear translocation,^62^ they do suggest that IL‐2 has a transcriptional effect independent of these pathways. Our data also show that while increasing GC concentration resulted in more suppression there was always more IL‐13 in high vs low IL‐2 conditions, suggesting that simply increasing GC dose in patients with high IL‐2 may not be effective. Indeed, McKeever et al^63^ reported that asthmatics receiving quadruple their GC dose still exhibited a 45% exacerbation rate in the following year compared to 52% in those that maintained usual GC dose. Our findings indicate this approach may be more effective if focused on patients who are “low IL‐2 producers,” that is, those with a potentially better chance of responding to increased GC.

The strong, super‐physiologic activation with PMA and ionomycin resulted in high production of IL‐2 from the patients’ peripheral blood cells in 1 mL of blood. This method has been published by others studying functional human immune responses,^32^, ^33^, ^34^ and showed a similar degree of variability in IL‐2 and tumor necrosis factor α production, whether cytokines levels were corrected for total cell numbers or not.^34^ In our in vitro system, we empirically developed a model of high and low IL‐2 based on typical concentrations used for Th2 cell cultures^10^, ^37^ as well as functional readouts of resistance/susceptibility to GC‐induced apoptosis. Concentrations were lower than observed after ex vivo mitogenic activation of patients’ cells, but similar to levels found to be produced from in vivo antigen‐activated T cells^64^ and BAL lymphocytes activated using beads coated with αCD2/CD3/CD28^13^ and so may be more physiologically relevant. The source of IL‐2 within whole blood could be naïve and/or memory CD4^+^ T cells,^64^ Th1 cells,^4^ CD8^+^ T cells^65^ or even dendritic cells previously exposure to Gram‐negative bacteria.^66^ Dendritic cell production would suggest prior infections may influence one's propensity to produce IL‐2 and could even occur in utero, as IL‐2 promoter methylation at birth was associated with asthma severity in childhood.^67^ IL‐2 genetic variants are also associated with asthma^68^, ^69^ as well as inflammatory bowel disease, type 1 diabetes and multiple sclerosis.^68^, ^70^, ^71^ Together, these data suggest that control of IL‐2, whether genetic or epigenetic, may be fundamental to the development of immune disease.

Our finding that suppression of IL‐13 required continual exposure to GC, since mRNA levels were double if DEX was received only as a pretreatment and not also during activation. These data suggest that unless (a) the dose of GC is sufficient to induce Th2 cell apoptosis and (b) exposure to GC is continual, suppression of IL‐13 is temporary. This could be important clinically since asthma phenotyping efforts recently proposed a classification based on type 2 cytokine expression.^72^ However, if a patient is taking a GC dose sufficient to suppress cytokines, but insufficient to eliminate Th2 cells, this could result in misclassification of type 2‐low asthma. Using a cell‐based approach, such as eosinophils and/or Th2 cells to classify type 2‐high asthma may improve identification of this phenotype. Further, it may help identify responders to new therapies that directly target type‐2 cells and their development, such agents blocking CRTh2 and TSLP.^2^, ^73^, ^74^

In summary, our study shows for the first time that IL‐2 reduces GC responsiveness of human Th2 cells by downregulating GR expression, supporting both their survival and pro‐inflammatory capacity in the face of GC treatment. These results suggest that a patient's potential to produce IL‐2 may be a determinant in asthma severity and that measuring peripheral levels of IL‐2 could help identify those in need of increased ICS dose and/or alternative therapies.
